# Understanding Public Perceptions and Discussions on Opioids Through Twitter: Cross-Sectional Infodemiology Study

**DOI:** 10.2196/50013

**Published:** 2023-10-31

**Authors:** Federico Carabot, Oscar Fraile-Martínez, Carolina Donat-Vargas, Javier Santoma, Cielo Garcia-Montero, Mariana Pinto da Costa, Rosa M Molina-Ruiz, Miguel A Ortega, Melchor Alvarez-Mon, Miguel Angel Alvarez-Mon

**Affiliations:** 1 Department of Medicine and Medical Specialities University of Alcala Alcala de Henares Spain; 2 Ramón y Cajal Institute of Sanitary Research Madrid Spain; 3 Department of Medicine and Medical Specialities University of Alcala Alcala de Henares, Madrid Spain; 4 Institute for Global Health Barcelona Spain; 5 Centro de Investigación Biomédica en Red | Epidemiología y Salud Pública (CIBER) Epidemiología y Salud Pública Madrid Spain; 6 Cardiovascular and Nutritional Epidemiology Unit of Institute of Environmental Medicine Karolinska Institute Stockholm Sweden; 7 Filament Consultancy Group London United Kingdom; 8 South London and Maudsley NHS Foundation Trust London United Kingdom; 9 Institute of Psychiatry, Psychology & Neuroscience King's College London London United Kingdom; 10 Department of Psychiatry and Mental Health San Carlos Clinical University Hospital, IdiSSC Madrid Spain; 11 Immune System Diseases-Rheumatology and Internal Medicine Service University Hospital Príncipe de Asturias Centro de Investigación Biomédica en Red | Enfermedades Hepáticas y Digestivas (CIBEREHD) Alcalá de Henares Spain; 12 Department of Psychiatry and Mental Health Hospital Universitario Infanta Leonor Madrid Spain

**Keywords:** awareness, epidemic, fentanyl, health communication, infodemiology, machine learning, opioids, recreational use, social media listening, Twitter, user

## Abstract

**Background:**

Opioids are used for the treatment of refractory pain, but their inappropriate use has detrimental consequences for health. Understanding the current experiences and perceptions of patients in a spontaneous and colloquial environment regarding the key drugs involved in the opioid crisis is of utmost significance.

**Objective:**

The study aims to analyze Twitter content related to opioids, with objectives including characterizing users participating in these conversations, identifying prevalent topics and gauging public perception, assessing opinions on drug efficacy and tolerability, and detecting discussions related to drug dispensing, prescription, or acquisition.

**Methods:**

In this cross-sectional study, we gathered public tweets concerning major opioids posted in English or Spanish between January 1, 2019, and December 31, 2020. A total of 256,218 tweets were collected. Approximately 27% (69,222/256,218) were excluded. Subsequently, 7000 tweets were subjected to manual analysis based on a codebook developed by the researchers. The remaining databases underwent analysis using machine learning classifiers. In the codebook, the type of user was the initial classification domain. We differentiated between patients, family members and friends, health care professionals, and institutions. Next, a distinction was made between medical and nonmedical content. If it was medical in nature, we classified it according to whether it referred to the drug’s efficacy or adverse effects. In nonmedical content tweets, we analyzed whether the content referred to management issues (eg, pharmacy dispensation, medical appointment prescriptions, commercial advertisements, or legal aspects) or the trivialization of the drug.

**Results:**

Among the entire array of scrutinized pharmaceuticals, fentanyl emerged as the predominant subject, featuring in 27% (39,997/148,335 posts) of the tweets. Concerning user categorization, roughly 70% (101,259/148,335) were classified as patients. Nevertheless, tweets posted by health care professionals obtained the highest number of retweets (37/16,956, 0.2% of their posts received over 100 retweets). We found statistically significant differences in the distribution concerning efficacy and side effects among distinct drug categories (*P*<.001). Nearly 60% (84,401/148,335) of the posts were devoted to nonmedical subjects. Within this category, legal facets and recreational use surfaced as the most prevalent themes, while in the medical discourse, efficacy constituted the most frequent topic, with over 90% (45,621/48,777) of instances characterizing it as poor or null. The opioid with the greatest proportion of tweets concerning legal considerations was fentanyl. Furthermore, fentanyl was the drug most frequently offered for sale on Twitter, while methadone generated the most tweets about pharmacy delivery.

**Conclusions:**

The opioid crisis is present on social media, where tweets discuss legal and recreational use. Opioid users are the most active participants, prioritizing medication efficacy over side effects. Surprisingly, health care professionals generate the most engagement, indicating their positive reception. Authorities must monitor web-based opioid discussions to detect illicit acquisitions and recreational use.

## Introduction

Chronic pain represents one of the leading causes of disability and disease burden worldwide [1,2]. Approximately 20%-30% of individuals have chronic pain globally [3,4]. In the United States alone, it costs between US $560 and US $635 billion per year [5-7]. Due to its complex nature, it represents one of the most significant medical challenges, involving numerous diagnostic and therapeutic difficulties [8].

Regarding the pharmacological management of chronic pain, opioids have traditionally been considered significant drugs for these patients. While there are few doubts about their use in acute pain, their prescription for chronic pain is often debated due to multiple long-term side effects, such as tolerance and dependency [9,10]. These facts, combined with overprescription and misuse, have contributed to a global health concern known as the opioid crisis [11]. Indeed, this opioid crisis has been responsible for the deaths of half a million people due to overdose in the United States, with nearly 70,000 deaths in 2020 alone [12].

Nowadays, social media holds immense sway, with over 58% of the global population actively engaged, offering researchers insights into health determinants through shared lifestyles, habits, and experiences. Social media’s impact extends to medical research, offering rapid and global insights on clinically significant topics. Real-time data collection aids studies on influenza spread, suicide risks, or substance abuse [13-18]. In fact, social media’s spontaneity offers an advantage over traditional methods, aiding in detailed patient insights [19,20]. Moreover, the use of social media data has proven useful as a pharmacovigilance tool [21-24]. In addition, it has proven to be effective in detecting underreported adverse effects [25,26]. For example, it is estimated that by monitoring the content published on social media, the thalidomide disaster could have been detected in 7 days [26]. Furthermore, previous studies have shown that it can also help monitor opioid crises by providing relevant information at a temporal or spatial level or even estimating mortality [13,27,28]. Locating inappropriate drug use plays a pivotal role in safeguarding public health. In this regard, analyzing posts made on social media can help identify medications that are being inappropriately acquired and used without a medical prescription and beyond pharmaceutical dispensation [13].

In this study, we aim to analyze content posted on Twitter related to major opioids with the following objectives: (1) characterize the users participating in these conversations; (2) identify the most frequently discussed topics, as well as understand the public’s perception of these drugs and the health care crisis surrounding them; (3) analyze Twitter users’ opinions regarding the efficacy and tolerability of these drugs; and (4) identify whether topics related to the dispensing, prescription, or acquisition of the drug are being discussed on Twitter.

## Methods

### Data Collection and Content Analysis Process

This analysis focused on tweets related to major opioids. We developed a list of 85 keywords (Textbox 1) referring to the brand names and generic names of the 7 most relevant major opioids: morphine, fentanyl, oxycodone, methadone, buprenorphine, tapentadol, and hydromorphone. We collected all tweets that met the following inclusion criteria: (1) public tweets; (2) including at least one of the selected keywords; (3) posted between January 1, 2019, and December 31, 2020; and (4) written in English or Spanish. We used Tweet Binder, a widely used research tool for comprehensive tweet search that provides access to 100% of public tweets [29-31].

A total of 256,218 tweets were collected, of which 69,222 were discarded. Two analysts examined a sample of 1000 tweets from each category, gathering annotations. These annotations were combined with insights from previous research and discussed in weekly meetings led and supervised by a senior researcher. These tweets were not manually classified but were part of the machine learning process. Subsequently, 7000 tweets (1000 from each drug) were analyzed according to a codebook created by the researchers specifically for this study. In the codebook, the type of user was the initial classification domain. We differentiated between patients, family members and friends, health care professionals, and institutions. This differentiation was based on both the content of the publication and the pronoun used by the user. Next, a distinction was made between medical and nonmedical content. If it was medical, we classified it according to whether it referred to the drug’s efficacy or adverse effects. Management was subcategorized based on whether the content of the publication referred to pharmacy dispensation, medical prescription, commercial aspects, or legal aspects. On the other hand, the classification of drug trivialization included humorous-jocular comments, references to recreational use, or the appearance of the drug in popular culture, such as song lyrics, essay chapters, or poetry. Subsequently, the remaining databases were then analyzed using machine learning classifiers.

**List of keywords.**

**Keywords**

1. Morfina, morphine, morfina clorhidrato, mst continus, oramorph, sevredol, zomorph, dolq, arymo, avinza, kadian, morphabond, ms contin, oramorph, roxanol.
2. Fentanilo, fentanyl, abfentiq, abstral, avaric, kaptic, actiq, effentora, breakyl, doloxital, durfenta, durogesic, fendivia, matrifen, instanyl, pecfent, fentora, onsolis.
3. Oxicodona, oxycodone, duoxona, nolxado, oxycontin, oxynorm, taioma, tanonalla, targin, dazidox, endocodone, eth-oxydose, oxaydo, oxecta, oxycontin, oxyfast, oxyir, percolone, roxicodone, xtampza.
4. Metadona, methadone, eptadone, metasedin, misyo, diskets, dolophine, methadose, westadone.
5. Buprenorfina, buprenorphine, buprex, feliben, transtec, belbuca, butrans.
6. Tapentadol, palexia, yantil, nucynta.
7. Hidromorfona, hydromorphone, edunix, jurnista, palladone, dilaudid, exalgo.

### Multilingual Machine Learning Classifier

In this project, we used a pretrained neural network known as XLM-RoBERTa. This model has been trained on an extensive data set comprising text in 100 different languages. XLM-RoBERTa is well-equipped to effectively process and comprehend multilingual text. Its popularity has been substantiated by over 13,000,000 monthly uses on Hugging Face, a prominent platform for deep learning models. This impressive usage rate solidifies its position as one of the most extensively used networks globally. The widespread adoption of XLM-RoBERTa highlights its efficiency and dependability across diverse applications.

To tailor the neural network to our specific classification needs, we executed a process known as fine-tuning. In this fine-tuning process, we retrained the neural network using our manually labeled data. For this purpose, we used 6000 manually classified tweets to retrain the network. Additionally, a set of 1000 manually classified tweets was used to assess the model’s performance. We used the *F*_1_ score as an evaluation metric to determine the classifiers’ precision. The *F*_1_ score is computed as the mean of precision and recall. After fine-tuning, all classifiers yielded *F*_1_ scores within the range of 0.75-0.8, indicating a good level of performance.

### Statistical Analysis

We estimated tweet frequencies by several characteristics of the tweets and the type of opioid. Comparisons of proportions were carried out using the Pearson chi-square test. Likewise, the number of retweets and likes between categories and opioids was compared using the ANOVA. A *P* value less than .05 was considered statistically significant. Analyses were conducted with the software packages STATA version 16 (StataCorp) and MS Excel (Microsoft Corporation).

### Ethical Considerations

This study was approved by the ethics committee of the University of Alcalá (OE 14_2020) and is compliant with the research ethics principles of the Declaration of Helsinki (seventh revision, 2013). This study did not directly involve human participants, nor did it include any intervention; instead, it uses only publicly available tweets (subject to universal access through the internet according to the Terms of Service that all users on Twitter accept). Nevertheless, we have taken care to not directly reveal in this report any username, and we have avoided citing tweets that could be offensive or compromised to someone.

## Results

### The Users Who Are Most Engaged in Conversations About Major Opioids Are Patients

Of all the drugs studied, fentanyl accumulated the highest percentage of tweets (Table 1). Specifically, 39,997 (27%) of 148,335 tweets were about fentanyl. Morphine, oxycodone, methadone, and buprenorphine represented between 16% (25,033/148,335) and 20% (29,330/148,335) of the tweets each. It is noteworthy that hydromorphone and tapentadol had a minimal presence, being mentioned in only 9% (13,466/148,335) and 1% (1788/148,335) of the tweets, respectively. All the drugs generated similar interest among Twitter users, except for tapentadol, which generated very little interest.

Regarding the user variable, greater differences were observed between different types of users (Table 1). Nearly 70% (101,259/148,335) of the users who posted tweets about these drugs were patients, while the remaining 30% (47,076/148,335) were distributed fairly evenly among family members or friends of patients, health care professionals, and health care institutions. Tweets from health care professionals received the most likes from other users. On the other hand, tweets published by health care institutions generated the least interest.

In terms of content, nonmedical content prevailed over medical content (Table 1). Nonmedical tweets predominantly addressed legal issues and the recreational use of these drugs. A total of 76% (48,777/63,934) of the medical content tweets discussed the topic of efficacy, and of these, over 90% (45,621/48,777) referred to null or low efficacy. Specifically, 48,777 tweets addressed efficacy, with 45,621 posts discussing poor or null efficacy.

**Table 1:** Descriptive characteristics of the original tweets included in the analysis, categorized by pain drug, type of user, type of content.

					Total, n (%)		Tweets with >100 likes, n (%)		Tweets with >100 retweets, n (%)
**Drug**					148,335 (100)		1022 (0.7)		365 (0.2)
	Morphine				27,368 (18.5)		187 (0.7)		105 (0.4)
	Fentanyl				39,997 (27**)**		333 (0.8)		161 (0.4)
	Oxycodone				28,061 (18.9)		172 (0.6)		73 (0.3)
	Methadone				29,330 (19.8)		170 (0.6)		20 (0.1)
	Buprenorphine				25,033 (16.9)		177 (0.7)		27 (0.1)
	Tapentadol				1788 (1.2)		2 (0.1)		0 (0)
	Hydromorphone				13,466 (9.1)		104 (0.8)		11 (0.1)
**User**									
	Patient				101,259 (68.3)		700 (0.7)		240 (0.2)
	Family or friend				17,934 (12.1)		124 (0.7)		79 (0.4)
	Health care professional				16,956 (11.4)		167 (1)		37 (0.2)
	Health institution				12,186 (8.2)		31 (0.3)		9 (0.1)
**Content**									
	**No medical content**				84,401 (56.9)		628 (0.7)		300 (0.4)
		**Management**		63,860 (75.7)		507 (0.8)		234 (0.4)	
			Pharmacy delivery	9207 (14.4)		78 (0.8)		19 (0.2)	
			Medical appointment	10,266 (16.1)		78 (0.8)		18 (0.2)	
			Commercial advertising	11,584 (18.1)		44 (0.4)		22 (0.2)	
			Legal issues	32,803 (51.4)		307 (0.9)		175 (0.5)	
		**Trivialization**		38,832 (46)		292 (0.8)		122 (0.3)	
			Humor and joke	9438 (24.3)		68 (0.7)		10 (0.1)	
			Recreational use	25,306 (65.2)		202 (0.8)		104 (0.4)	
			Song, poetry, or book	4088(10.5)		22 (0.5)		8 (0.2)	
	**Medical content**				63,934 (43.1)		394 (0.6)		65 (0.1)
		**Refer to efficacy**		48,777 (76.2)		302 (0.6)		36 (0.1)	
			None or little efficacy	45,621 (71.3)		287 (0.6)		34 (0.1)	
			Good efficacy	3156 (4.9)		15 (0.5)		2 (0.1)	
		Refer to side effects		35,021 (54.7)		200 (0.6)		46 (0.1)	

### Health Care Professionals Primarily Focus Their Tweets on Buprenorphine, While Patients Center Their Discussions Around Fentanyl and Oxycodone

**Figure 1:**
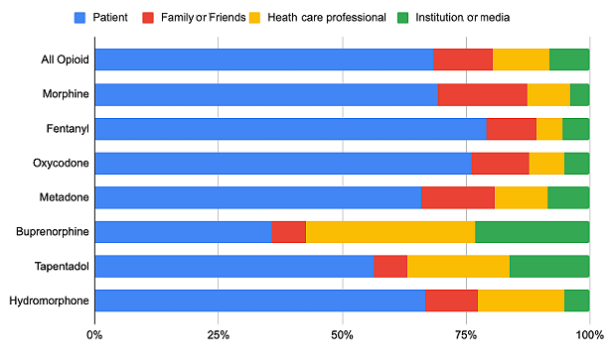
Number of tweets posted for each drug according to the type of user.

We found statistically significant differences in the distribution of tweets among different types of users for the various drugs (*P*<.001). Health care professionals have tweeted mostly about buprenorphine, tapentadol, and hydromorphone, while health care institutions have focused primarily on buprenorphine, tapentadol, and hydromorphone (Figure 1). On the other hand, family members have focused on morphine and methadone. Finally, users identified as patients have centered their discussions around fentanyl and oxycodone.

### Twitter Users Perceive Major Opioids as Being of Low Efficacy

We also found statistically significant differences in the distribution of tweets regarding efficacy and side effects among the different types of drugs (*P*<.001). Approximately 40%-50% of the tweets mentioning morphine, buprenorphine, tapentadol, or hydromorphone referred to the null or low efficacy of the drug (Table 2). For oxycodone and methadone, this percentage was lower, and for fentanyl, only 13% (5359/39,997) of the tweets mentioned null or low efficacy. On the other hand, the drug that accumulated the highest percentage of tweets with a favorable opinion of efficacy was hydromorphone (1087/13,466, 8.1%), followed by tapentadol (119/1788, 6.7%) and morphine (1004/27,368, 3.7%). As for side effects, all drugs received a similar percentage of tweets, with buprenorphine being the least tolerated and fentanyl being the best tolerated.

**Table 2:** The number of tweets discussing the efficacy of each drug and the presence of side effects.

		Tweets about efficacy, n (%)			Tweets about side effects, n (%)	Total
		None or little	Good			
**Drug**		45,621 (30.8)	3156 (2.1)	35,021 (23.6)		148,335
	Morphine	11,466 (41.9)	1004 (3.7)	6502 (23.8)		27,368
	Fentanyl	5359 (13.4)	367 (0.9)	6953 (17.4)		39,997
	Oxycodone	7970 (28.4)	518 (1.8)	7270 (25.9)		28,061
	Methadone	9620 (32.8)	353 (1.2)	6877 (23.4)		29,330
	Buprenorphine	11,138 (44.5)	387 (1.5)	8605 (34.4)		25,033
	Tapentadol	691 (38.6)	119 (6.7)	523 (29.3)		1788
	Hydromorphone	6478 (48.1)	1087 (8.1)	3815 (28.3)		13,466

### The Majority of Tweets About Fentanyl Discuss Legal Issues or Make References to Recreational Use

Within the nonmedical content, legal aspects and mentions of recreational drug use stood out (Table 3). The substances with the greatest proportion of tweets concerning legal considerations were fentanyl (constituting 18,048/39,997, 45.1% tweets out of a total of 32,803), followed by oxycodone (8375/28,061, 29.8%) and methadone (4864/29,330, 16.6%). Furthermore, fentanyl was the drug most frequently offered for sale on Twitter, as 17.7% (7075/39,997) of the tweets about this drug included references to its sale through the social network. Methadone generated the most tweets about pharmaceutical distribution, while buprenorphine generated the most tweets about medical prescriptions.

**Table 3:** Distribution of nonmedical content tweets in the different categories.

		Tweets about trivialization, n (%)					Tweets about management, n (%)				
		Total	Joke and humor	Recreational use	Songs, poetry, or books	Total		Delivery	Bureaucracy	Advertising	Legal
**Drug**		38,832 (26.2)	9438 (6.4)	25,306 (17.1)	4088 (2.8)	63,860 (43.1)		9207 (6.2)	10,266 (6.9)	11,584 (7.8)	32,803 (22.1)
	Morphine	5543 (20.3)	2562 (9.4)	1106 (4)	1875 (6.9)	5438 (19.0)		1280 (4.7)	1867 (6.8)	461 (1.7)	1830 (6.7)
	Fentanyl	22,494 (56.2)	1242 (3.1)	20,861 (52.2)	391 (1)	26,480 (66.2)		924 (2.3)	433 (1.1)	7075 (17.7)	18,048 (45.1)
	Oxycodone	3579 (12)	987 (3.5)	1889 (6.7)	703 (2.5)	13,016 (46.4)		1205 (4.3)	831 (3)	2605 (9.3)	8375 (29.8)
	Metadone	6924 (23.6)	4380 (14.9)	1547 (5.3)	997 (3.4)	10,729 (36.6)		3184 (10.9)	1998 (6.8)	683 (2.3)	4864 (16.6)
	Buprenorphine	326 (1.3)	161 (0.6)	114 (0.5)	51 (0.2)	11,202 (44.7)		2474 (9.9)	6093 (24.3)	1056 (4.2)	1579 (6.3)
	Tapentadol	36 (2)	18 (1)	17 (1)	1 (0.1)	424 (23.7)		101 (5.6)	51 (2.9)	194 (10.9)	78 (4.4)
	Hydromorphone	925 (6.9)	242 (1.8)	568 (4.2)	115 (0.9)	1916 (14.2)		1179 (8.8)	61 (0.5)	157 (1.2)	519 (3.9)

References to the recreational use of significant opioids were detected in approximately 20% (25,306/148,335) of the overall tweets. Fentanyl stood out in this regard, as over half of the tweets mentioning this drug referred to its recreational use. Additionally, approximately 15% (4380/29,330) of the tweets about methadone included jokes, while morphine was the most prevalent in popular culture (songs, poetry, or books).

## Discussion

### Overview

In this work, our primary findings indicated that the majority of the posts were authored by patients. It is noteworthy that users turn to Twitter to voice their discomfort and seek potential solutions, thus emphasizing their focus on the drug’s efficacy over potential side effects. This inclination could be perilous, among other reasons, as only a minority appear content with the analgesic efficacy. Another significant finding was the predominant mention of fentanyl and methadone, underscoring the current concern regarding the opioid crisis and its most frequently implicated medications. In a previous study that analyzed Twitter content related to opioids, it was also found that the majority of the posts came from individuals sharing their personal experiences [32]. However, our results show that, despite the relatively low presence of health care compared to those considered patients, they obtained the highest number of retweets and interest from Twitter users. Other studies have also found that when the author was a medical expert, people were more likely to believe the content they shared with web-based sources [33]. Moreover, previous studies have claimed that social media provide a unique opportunity for health care professionals to engage with the community, representing a potential opportunity for accelerating knowledge transmission [34]. Based on these results, we strongly encourage a greater presence of health professionals on web-based resources such as Twitter to raise awareness and inform people about the risks associated with opioids.

Fentanyl is a synthetic opioid introduced more than 60 years ago, being 50-100 times more potent than morphine [35]. The use of this drug has been rapidly expanded since the early 1990s due to the availability of different forms of administration along with its potency, which made it a widely accepted resource for doctors and patients [36]. However, inappropriate prescriptions by clinicians and increased illicit use and abuse were responsible for an important number of deaths and problems related to its abuse [37,38]. Likewise, oxycodone presents certain pharmacological characteristics that contribute to its high likability and abuse susceptibility [39], which favors its illicit nonmedical nature to the point of becoming a problem [40]. Thus, it is understandable that both fentanyl and oxycodone were the most common major opioids discussed by patients, due to the uses and impact that they may have on addiction and other concerns.

Morphine and methadone were more frequently tweeted by family and friends. The reason why family and friends tweet about morphine can be explained due to the extended knowledge about this drug, its use in palliative care, and its presence in popular culture (songs, poetry, or books). On the other hand, previous research conducted on Twitter has found that in general, both buprenorphine and methadone share the same major themes [41], although another study found that buprenorphine was more commonly commented as a medication against opioid use disorder (OUD) than methadone [42]. Our observations that health care professionals or institutions share more information about buprenorphine can be a plausible explanation for the differences observed in previous works. In the event of the past 2 major opioids discussed, hydromorphone and tapentadol, we highlight that they were more commonly tweeted by health care professionals or institutions, which can be explained by the specific uses and potential side effects of both opioids in clinical practice [43,44]. Nevertheless, tapentadol was the major opioid that generated less interest. The differences in the number of tweets containing tapentadol with other major opioids have also been found previously [45]. However, the use and illicit sale of this drug must also be surveilled.

Regarding the content of tweets, we observed that nonmedical content was superior to medical concerns. In more detail, we observed that nonmedical tweets mainly discussed legal issues and recreational uses (in which the majority of them were treating drug trivialization). Of the different major opioids analyzed, fentanyl accumulated the most tweets. Fentanyl stood out in recreational uses, as more than 50% (20,861/39,997) of the tweets containing this opioid discussed this topic, also representing the drug with the highest percentage of tweets referring to legal aspects, followed by oxycodone and methadone. In agreement with our results, previous works evaluating web-based posts found that fentanyl misuse, overdose, and death have dominated discussion in recent years over other types of drugs [46]. Indeed, according to the Centers for Disease Control and Prevention (CDC), the opioid crisis presented 3 waves of overdose deaths: the first wave starting in the 1990s related to increased prescribing of opioids; the second wave between 2010 and 2013, with rapid increases in overdose deaths associated with heroin; and the third wave, with significant raises in overdose deaths involving synthetic opioids, particularly those involving illicitly manufactured fentanyl [47]. Thus, the number and causes of deaths have evolved since the start of the opioid crisis, and fentanyl has been at the center of the problem since the beginning of the third wave. Recreational uses of fentanyl are frequently consumed from 2 major sources. First, and most commonly, it combines illicitly manufactured fentanyl from clandestine sources, often mixed up with heroin (“fake heroin”) to increase its potency at a low cost or included in cocaine products. In other cases, it can also be mixed into and sold as oxycodone-, hydrocodone-, or alprazolam-containing tablets [48]. The recreational use of fentanyl is a notable issue to address nowadays, as it is estimated that this opioid is involved in 71% of all drug overdose deaths, especially in combination with cocaine and heroin [49]. It is of great concern that most tweets treating recreational uses of fentanyl were focused on trivialization. Previous works have also found that despite widespread awareness of the risks of fentanyl, people who use it feel that the risks of having an overdose from its users tend to be perceived as null or low, particularly the longer they have been consuming it [50]. Similarly, the use of fentanyl as an illicit drug has gained preference in the past years [48,51], whereas recent works conducted on Twitter and other social media found evidence of ongoing illicit and controlled opioid drug promotion [52,53]. The fact that an important subset of tweets treats the trivialization of opioids and fentanyl as recreational drugs clearly reveals the lack of awareness and concern in our society about the dangers and the state of the art of the opioid crisis, which is a major global problem to face with a substantial impact even nowadays.

Simultaneously, we have found that 17.7% (7075/39,997) of tweets about this drug referred to its sale through social networks. In this line, previous works conducted in the Twittersphere have detected a significant number of tweets marketing the sale of fentanyl on the internet illicitly, and some of them contained hyperlinks to external websites, including web-based fentanyl advertisements and illicit web-based pharmacies [54]. Interestingly, the general use of Twitter as an illegal web-based sale of other opioids like oxycodone and codeine has also been reported in previous works [55,56]. Thus, as suggested and supported by previous works, Twitter represents a potential conduit for the supply and sale of illicit fentanyl and other opioids, and further measures and solutions should be directed in this field. In this context, it is also understandable that many tweets consider legal questions about the use of opioids, particularly in the case of fentanyl. Legal questions around the use of opioids undoubtedly represent one of the most important issues to face in terms of ensuring adequate use of opioids in clinical practice and limiting the impact caused by the opioid crisis. In the United States, the CDC, Food and Drug Administration, the Drug Enforcement Agency, and the Department of Justice collaborate in the opioid crisis epidemic, giving each institution a defined role [57]. However, even though efforts are made, the rates of opioid misuse and nonfatal and fatal overdose remain high. Thus, previous works claim that further strategies are strongly warranted to facilitate the implementation and enforcement of laws that apply only to specific subsets of providers, patients, or prescriptions and address issues of access and data use in the prescription drug monitoring program [58,59]. Thus, it is not surprising that a significant number of tweets open a debate about legal questions and possible measures to counteract the impact of opioid misuse worldwide.

On the other hand, most medical content on Twitter about opioids claimed low efficacy from their use (45,621/48,777, 90% of the tweets classified in this field reported null or low efficacy). Available scientific evidence shows a high heterogeneity in the effectiveness and benefits of opioid administration in the management of chronic pain. For instance, some studies have failed to find any significant effect of major opioids on pain relief, functional outcomes, and quality of life of patients receiving this drug [60]. Other studies report, however, that opioids (especially major opioids) outperformed placebos for pain and function in all types of noncancer chronic pain and that some patients benefit from their use [61]. However, they have also found that other drugs performed better functional outcomes than opioids, especially at long-term therapy (≥6 months), when it seems to be associated with more adverse events, opioid abuse, or dependence, and possibly an increase in all-cause mortality. Besides, the lack of clinically meaningful predictive biomarkers and the fact that an important percentage of patients (>33%) tend to withdraw clinical interventions after opioid therapy promote controversy over the effectiveness and applications of these drugs [62,63]. Nevertheless, there are also studies that have identified that an important subset (92%) of clinicians and patients believe that opioids reduce pain and, to a lesser extent (57%), that their use has been associated with improvements in their quality of life [64]. This study is contrary to these perceptions, especially for morphine, tapentadol hydromorphone, and buprenorphine, and to a lesser extent in the case of oxycodone, methadone, and fentanyl. It was also of note that buprenorphine was the worst-tolerated opioid and fentanyl the best-tolerated, as only 13% of the tweets referred to null or low efficacy. Previous works conducted on Twitter have reported little difference in the perceived efficacy of different drugs, as is the case with buprenorphine and methadone [41]. In this sense, this study gains further insights into the opinions of patients receiving medical treatment with various opioids. It is important to consider the opinions and sentiments of people who use opioids on Twitter, as it can represent a medium in which the patient can express their feelings about a drug freely without feeling judged or devalued [24]. Besides, previous scientific studies revealed that social network support may have a favorable effect on the therapeutic success of OUD [65], demonstrating the relevance of these platforms in improving patients’ odds of recovery from OUD and reversing the rising trend in opioid deaths. Conversely, in our results, we report the negative opinion of most Twitter users regarding the benefits of opioids, which could indicate an imperative need to improve medical uses of opioids and reassess the efficacy of the selected treatments in each patient.

### Limitations

This study has some limitations. First, the social, economic, and demographic characteristics of Twitter users are not a true reflection of society. Second, the design of the codebook and the analysis of the tweets entail, like almost all qualitative studies, a certain subjectivity. Third, we may have missed tweets that referred to stronger opioids but used different words, such as slang or contractions.

### Conclusions

The opioid crisis is present on social media, with discussions encompassing both legal and recreational use. Opioid users are the most active participants, and their conversations predominantly center on drug efficacy, often considering it low rather than focusing on potential side effects. This finding suggests that the general population, particularly opioid users, may underestimate the risks associated with opioid consumption and could have developed a tolerance to its effects. Hence, there is a critical need for health care professionals to disseminate appropriate medical information about opioids on platforms like Twitter. Additionally, addressing these topics during medical consultations is essential. It is also concerning that social media is being used to promote recreational opioid use or even illicit acquisition. Therefore, authorities must closely monitor web-based discussions related to opioids to detect illegal acquisitions or recreational use.

## Abbreviations

CDC: Centers for Disease Control and Prevention
OUD: opioid use disorder

## Data Availability

The data sets generated or analyzed during this study are available from the corresponding author on reasonable request.
